# Towards optimal morphology in organic solar cells?

**DOI:** 10.1093/nsr/nwaf563

**Published:** 2025-12-13

**Authors:** Oskar J Sandberg, Ronald Österbacka

**Affiliations:** Faculty of Science and Engineering, Åbo Akademi University, Finland; Faculty of Science and Engineering, Åbo Akademi University, Finland

Organic solar cells (OSCs) show great promise within the renewable energy sector, as sustainable and cost-effective alternatives comprising lightweight and flexible solar cells manufactured using low-cost methods such as printing [1]. However, in contrast to other solar cell technologies, OSCs are inherently excitonic, meaning that the photon absorption produces strongly bound excitons rather than free charge carriers. To overcome this, OSCs based on bulk heterojunction (BHJ) blends of two different organic semiconductor components—a donor (D) and an acceptor (A)—are commonly used. Excitons are efficiently dissociated at the D–A interfaces, ultimately generating free electrons (holes) in the acceptor (donor) phase. The free electrons (holes) are subsequently transported in the acceptor (donor) phase to the cathode (anode) where they are collected [2]. The phase separation in the BHJ needs to be optimized to maximize the exciton dissociation, while simultaneously ensuring percolating, continuous transport pathways for charge extraction. As such, controlling the morphology in OSCs is of utmost importance, complicated by the fact that the morphology in state-of-the-art BHJs is generally not stable, leading to poor overall stability [3].

**Figure 1.**
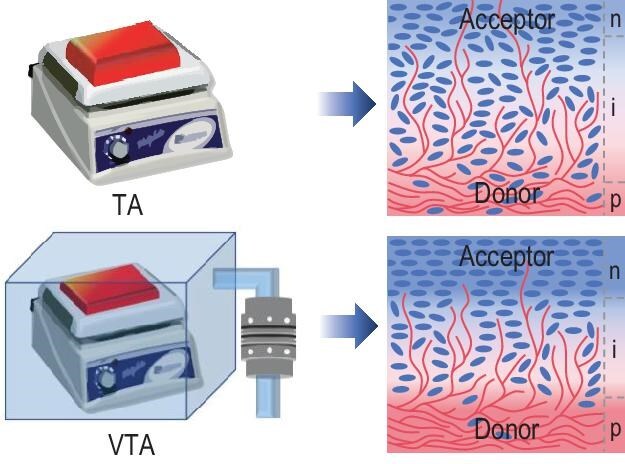
Schematics of the morphology of BHJs achieved by TA and VTA. Using the VTA process, an improved p-BHJ morphology can be achieved. Reproduced with permission from Cheng *et al*. [5].

The stability can, in principle, be increased using a planar heterojunction (PHJ) where a donor and acceptor layer is stacked one upon the other. While PHJs facilitate selective charge extraction, the exciton dissociation is limited to one exciton diffusion length (typically around 10 nm) from the D–A interface, resulting in a substantial loss in photocurrent. An alternative approach that combines the advantages of BHJs and PHJs is to use a pseudo-BHJ (p-BHJ) structure (see Fig. 1, right). The p-BHJ is constructed by sequential deposition of donor and acceptor, intermixing and forming continuous transport pathways through vertical compositional gradients. Ideally, the p-BHJ forms a *p-i-n*-like structure, with donor- and acceptor-rich domains at the anode and cathode, and a gradually more intermixed morphology in between [4]. However, achieving an ideal morphology has remained challenging.

To improve the morphology of OSCs, post treatments such as thermal annealing (TA) are commonly employed. In the paper by Cheng and co-workers, they introduce a post-treatment based on vacuum-assisted TA (VTA) that combines vacuum and thermal annealing (see Fig. 1) [5]. The VTA treatment was applied to p-BHJ OSCs, showing superior performance and stability compared to standard TA. VTA-treated p-BHJs based on D18:L8-BO and PM6:L8-BO reached impressive power conversion efficiencies of 20.5% and 20.0%, respectively. The enhancements were underpinned by increases in short-circuit current densities, fill factors and open-circuit voltages, consistent with improved charge carrier generation, extraction and selectivity.

VTA accelerates solvent evaporation, enhances acceptor crystallization at the top surface, and reduces excessive intermixing of donor and acceptor materials. As a result, a more stratified configuration, resembling an optimal *p-i-n*-like morphology, is obtained. Techniques probing the morphology further revealed enhanced crystallinity and molecular packing in VTA-treated films. The VTA treatment was also found to significantly reduce the burn-in effect, resulting in enhanced operational stability of the OSCs. VTA-treated OSCs exhibited T80 lifetimes exceeding 3900 h under ISOS-L-1 conditions and 5400 h when using a device structure with stable contacts. Furthermore, VTA-treated OSCs showed improved resilience against thermal, light and moisture stresses, as evidenced by T80 lifetimes of over 1200 h achieved under accelerated ISOS-L-3 conditions.

In summary, VTA post-treatment of p-BHJs appears to form the optimal morphology in OSCs, leading to higher efficiencies and improved stability due to better crystallization and suppressed intermixing, ultimately advancing OSC technology.
